# Incidence and root causes of medication errors by anesthetists: a multicenter web-based survey from 8 teaching hospitals in Ethiopia

**DOI:** 10.1186/s13037-023-00367-8

**Published:** 2023-06-15

**Authors:** Meseret Firde

**Affiliations:** Department of anesthesia, Debre Tabor University, Po.box: 272, Debre Tabor, Ethiopia

**Keywords:** Anesthetic drug, Medication admnistration, Drug admnistration, Medication admnistration error

## Abstract

**Bakground:**

The operating room is a demanding and time-constrained setting, in comparison to primary care settings, where perioperative medication administration is more complicated and there is a high risk that the patient will experience a medication error. Without consulting the pharmacist or seeking assistance from other staff members, anesthesia clinicians prepare, deliver, and monitor strong anesthetic drugs. The purpose of this study was to determine the Incidence and root causes of medication errors by anesthetists in Amhara region, Ethiopia.

**Methods:**

A multi-center cross sectional web-based survey study was conducted from October 1 to November 30, 2022, across eight referral and teaching hospitals of Amhara region. A self-administered semi structured questionnaire was distributed using survey planet. Data analysis was conducted using SPSS version 20. Descriptive statistics were computed and binary logistic regression was used for data analysis. A p-value < 0.05 was considered statistically significant.

**Results:**

The study included 108 anesthetists in total, yielding a response rate of 42.35%. Out of 104 anesthetists, Majority of participants (82.7%) were male. During their clinical practice, more than half (64.4%) of participants experienced atleast one drug administration error. 39 (37.50%) of the respondents revealed that they experienced more medication errors while on night shifts. Anesthetists who did not always double-check their anesthetic drugs before administration had a 3.51 higher risk of developing MAEs compared to those who always double-check anesthetic drugs before administration (AOR = 3.51; 95% CI: 1.34, 9.19). Additionally, participants who administer medications that have been prepared by someone else are about five times more likely to experience MAEs than participants who prepare their own anesthetic medications prior to administration (AOR = 4.95; 95% CI: 1.54, 15.95).

**Conclusion:**

The study found a considerable rate of errors in the administration of anaesthetic drugs. The failure to always double-check medications before administration and the use of drugs prepared by another anaesthetist were identified to be underlying root causes for drug administration errors.

## Background

The operating room is a demanding and time-constrained setting, in comparison to primary care settings, where perioperative medication administration is more complicated and there is a high risk that the patient will experience a medication error [1]. Without consulting the pharmacist or seeking assistance from other staff members, anesthesia clinicians prepare, deliver, and monitor strong anesthetic drugs [2, 3]. Studies in anesthesia have shown that drug errors have been highly prevalent globally [4, 5].

The range of consequences from medication error effects runs from no notable effects to death. In some cases, it can cause a new condition, either temporary or permanent, such as itching, rashes, or skin disfigurement [6, 7]. Inadvertently prescribing the incorrect medication to a patient or having a near-miss could cause shame, guilt, and self-doubt in medical professionals. This is known as the “second victim,“ and it can have potentially fatal consequences [8]. In addition to suing the healthcare provider, patients or patients’ families may also sue the healthcare facility where the healthcare provider works for personal injury [9–11]. An extensive self reported survey result conducted in Canada, shown that Although most errors (1,038) were of minor consequence (98%), four deaths were reported [12]. In addition to increasing the risk of patient morbidity and even death, incorrect drug administration can increase the length of hospital stays, which has an impact on the economy.

Furthermore, Hospitals may have to pay a significant amount for legal counsel and potential settlement costs. Along with the increased cost of unplanned prolonged hospitalisation and patient care, hospitals may also be responsible for the lost productivity of the staff involved in the error [13]. Dealing with the errors, investigation, litigation, and settlement may also take some time. The management team of the hospital may have to invest time and resources to look into and change policies to reduce future mistakes [5, 14]. Aside from causing personal harm, adverse drug reactions put a heavy financial burden on healthcare systems. Therefore, successful efforts to stop medication errors will increase patient safety and result in cost savings On the other hand it is documented that one of the greatest ways offered to improve patient safety by minimizing the volume of medication administraton errors (MAEs) is to identify and intervene with the underlying root causes of MAEs [9]. One of the least studied and unaddressed health issues in developing countries like Ethiopia, which have issues with their economies, workforce, and education, is MAEs in anesthetic clinical practice. As a result, the purpose of this study was to determine the type, how frequently and what underlying root causes lead to medication errors in patients under anesthesia.

## Methods

A multicenter web-based cross-sectional study design was conducted among anesthetists working at Amhara region in Ethiopia from October 1 to November 30, 2022. The study was carried out in eight teaching hospitals, including the Woldya Comprehensive Specialized Hospital, the Tibebe Gion Comprehensive Specialized Hospital, the Felege Hiwot Comprehensive Specialized Hospital, the Debremarkos Comprehensive Specialized Hospital, the Debrebrhan Comprehensive Specialized Hospital, and the Debretabor Comprehensive Specialized Hospital. Because of increasing patient flow and clinical practitioners, those referral and teaching institutions were chosen to better reflect actual clinical practice.

Utilizing Survey Planet and their email and telegram addresses, 255 anesthetists employed at the specified hospitals received the questionnaires. The inclusion Criteria were all anesthetists with a minimum of six months of working experience, the ability to respond to the questioner using the listed online choices, and involved in direct patient care to be included in the study. Those anesthetists who are not involved in medication administration practice and the ones serving in administrative positions were excluded from the study.

The dependent variables were Prevalence of MAEs and Socio-demographics characteristics, work-related factors, professional related factors, and other error producing conditions were among independent variables. For the data collection a semi-structured self-administered questionnaire was prepared and used to collect data on anesthetists’ socio-demographic characteristics (an institution where the anesthetists earned, an educational award, year of experience, etc.), work-related factors (presence of written guideline for medication administration, training on medication administration, communication with other anesthetists while facing problems and availability of reporting mechanism to medication errors), Moreover, the prevalence of MAE, reporting trends of a medication error, and types of MAE were considered. MAEs were defined as any error made during the administration of anesthetic drugs regardless of how many errors were made or whether there were any adverse consequence.

Five MSc-trained anesthetists who reside in or close to the study area were involved in the data collection process. Before real data collection, the questionnaire was pretested on 10 anesthetists employed at Dilla University Hospital in order to ensure the quality of the data. Before participants began the survey, we gave each participant a page of material outlining the study’s goals and the primary investigator’s contact information.

Given the nature of web-based questionnaires (absence of the researcher), respondents were motivated to answer more honestly because we maintained their anonymity. We secured the study participants’ information and upheld their confidentiality. To prevent response bias, which occurs when respondents lose interest and give answers that are consistent with the question, the survey questioner took between five and ten minutes to complete. For validation reasons, the gathered data were imported into Epidata version 4.2. Version 20 of the Statistical Package for Social Science (SPSS) was used to analyze the data).

As frequencies and percentages, the descriptive statistics’ findings for nominal categorical variables are enumerated. For statistical inference, we used nonparametric tests. In order to create charts, we used SPSS and Microsoft Excel.The study population was described with regard to pertinent factors using statistics including frequencies, proportions, and summary statistics (mean, median, IQR, and standard deviation), which were then displayed in tables and graphs. Finally, factors from the multivariable analysis were deemed statistically significant if their p-value was less than 0.05. To measure the degree of relationship between medication administration error and its predictors, an adjusted odds ratio with a 95% confidence interval (CI) was taken into account.

## Results

The study included 108 anesthetists in total, yielding a response rate of 42.35%. One hundred four were discovered to be complete and analayzed. The mean (standard deviation) age of anesthetists was 29.06(± 4.007) years. Male participants made up 82.7% of the total. Approximately 67 (64.4%) of the participants had over five years of work experience in clinical anesthesia. The majority of study participants 71 (68.3%) had a master’s degree in science (MSc) or higher (Table 1).

**Table 1:** Socio-demographic characteristics of participants in teaching hospitals of Amhara region, Ethiopia (n = 104)

Variables	Response	Frequency (n)	Percentage(%)
Gender	Male Female	86 18	82.7 17.3
Age	≤ 25 26–30 ≥ 31	24 34 46	23.1 32.7 44.2
Marital status	Single Married Divorced	36 61 7	34.6 58.7 6.7
Level of education	Bsc MSc	33 71	31.7 68.3
Work experiance	≤ 5 > 6	37 67	35.6 64.4

Regarding the characteristics of the participants’ working environments, more than two thirds of participants 90 (86.54%) worked exclusively in governmental hospitals. In addition to their clinical work, more than half of the respondents were involved in academic and teaching endeavors. The availability of medications at 85(81.73%) of the participants’ working hospitals was revealed to be insufficient or not continuous. However, during their clinical practice, 91 (87.50%) of the participants did not receive training on medication administration error. Additionally, only 30 (28.85%) of respondents indicated that their hospital has a working system for reporting medication errors (Table 2).

**Table 2:** Working area related characterstic of the participants (n = 104)

Variables	Response	Frequency (n)	Percentage(%)
**Working area**	Governmental hospital Private hospital Govermental and private hospital	90 7 7	86.54 6.73 6.73
**Practice speciality apart from anesthesia**	Only anesthesia practice Critical care Teaching/acadamics Adminstrative responsibility	17 22 56 98.7	16.35 21.15 53.85 8.65
**Active working duty shift**	Day shift Night shift Alternative shift	11 15 78	10.60 14.40 75.00
**working hours per day**	< 8 h > 8 h	49 55	47.10 52.90
**does your hospital anesthetic drug supply adequate**	yes no	19 85	18.27 81.73
**do you use colour coded syring for drug admnistration**	Yes No	3 101	2.90 97.11
**have you take medication adminstration training**	Yes No	13 91	12.50 87.50
**do you have medication adminstration guideline**	Yes No	33 71	31.73 68.30
**does your hospital has medication error reporting system**	Yes No I don’t know	30 56 18	28.85 53.80 17.31
**how often your department audit the critical incident registery**	Monthly Every year i dont know	3 15 86	2.90 14.42 82.70
**how many number of anesthetists involved per table during your usual practice**	1 2 3	23 78 3	22.11 75.00 2.90

In accordance with the findings, 43 (41.3%) of respondents admitted to using anesthetic medications that were prepared by students under supervision for their usual clinical practice. Additionally, 53 (51.00%) of the participants stated that they labeled the syringe first before withdrawing the drug when preparing the medication. The remaining participants (49.00%) admitted that they label the srying after withdrawing the anesthetic drugs. Before administering anesthetic medications, only 38 (36.5%) of those surveyed claimed to always double check, which entails two people making sure the same information is accurate. During their clinical practice, more than half (64.4%) of participants experienced drug administration error. 39 (37.50%) of the respondents revealed that they experienced more medication errors while on night shifts (Table 3).

**Table 3:** Process and characteristics of the participant’s drug administration (n = 104)

Variables	Response	Frequency (n)	Percentage(%)
**Who usually prepare anesthetic drug**	students under supervision another anesthetist your own	43 31 30	41.30 29.80 28.80
**how draw the drug into the srying**	label the syring first then withdraw the drug withdraw the drug then label the srying	53 51	51.00 49.00
**do you always double check drugs before admnistration**	Yes No	38 66	36.50 63.50
**do you faced interruption during medication administration**	Yes No	45 59	43.3 56.7
**when present with doubt do you communicate with another anesthetist**	yes no	37 67	35.57 64.42
**Have you faced drug adminstration error in your anesthesia practice**	yes no	67 37	64.40 35.60
**If yes, how often do you make medication adminstration error**	sometimes most of the time	60 7	57.69 6.73
**when have you experianced more incident of medication error**	daytime working hours night duty not related to time of work	10 39 18	9.62 37.50 17.31
**Have you ever reporting any drug error while reporting**	Never 1–4 reports reported 5–10 error reports	54 10 3	51.92 9.62 2,88
**when you commit medication adminstration error, how do you report it**	to my colleague i did not disclose any error	12 55	11.54 52.88
**if you are not report medication adminstration error, why**	fear of medicolegal issue not sure to whom it reported fear of jugment by colleagues	32 25 10	30.77 24.04 9.62

**Fig. 1:**
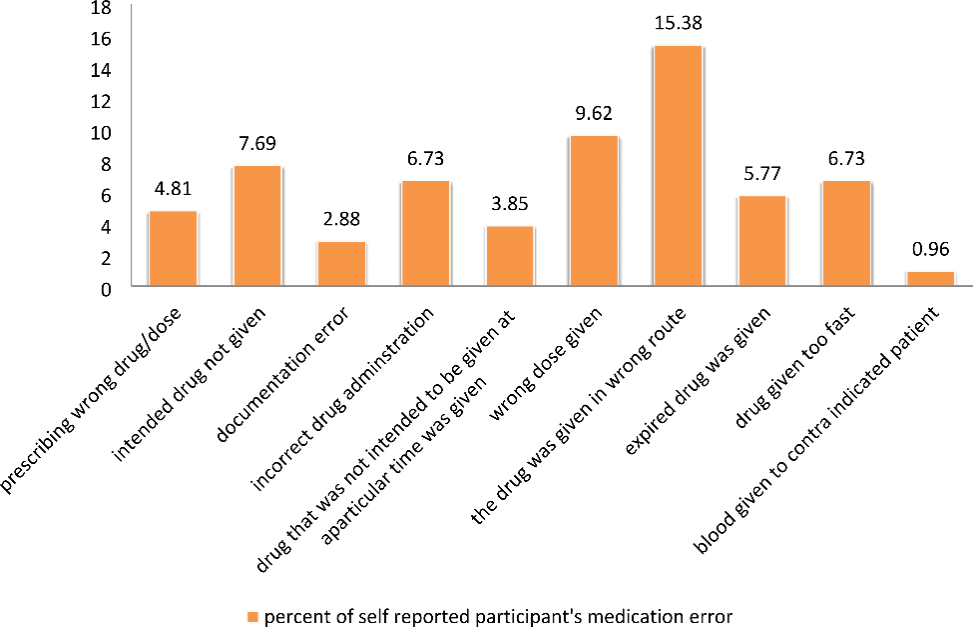
Type and percentage of self reported participants’ medication error

According to the finding of this study, giving drugs in wrong route were the frequent type of medication error reported by majority of participants who admitted MAEs (15.38%) followed by wrong dose (9.62%) and intended drug not given at aparticular time (7.69%) (Fig. 1).

**Fig. 2:**
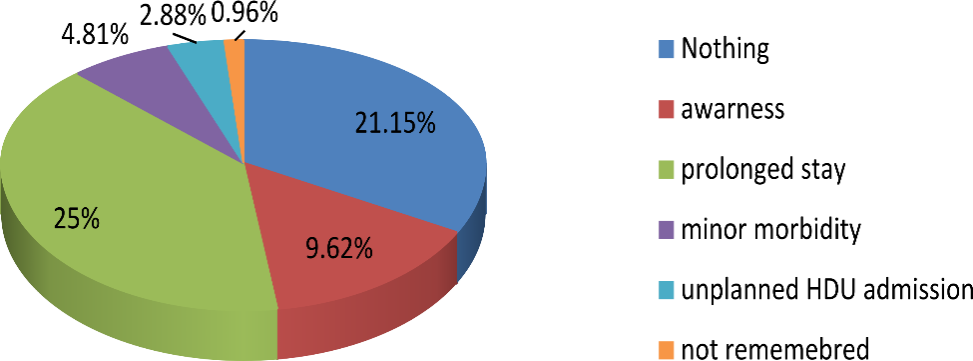
Type and percentage of injuries that witnessed by participants as a result of medication administration error

The more frequent complication of medication administration error observed by participants is prolonged hospital stay following surgery and anesthesia(25%) (Fig. 2).

### Underlying root causes of medication administration errors (MAEs)

In the bivariate analysis, the availability of the medication administration guideline, whether or not participants always double-check anesthetic drugs before administration, by whom typically anesthetic drugs prepared, how anesthetic drugs drown in the syringe, and whether or not anesthetists communicate with other anesthetists when they faced difficulties during the drug administration process were significantly associated with MAEs. However, the multivariable logistic regression model found a significant relationship between whether participants always double-check anesthetic drugs before administration and who typically prepares anesthetic drugs.

Anesthetists who did not always double-check their anesthetic drugs before administration had a 3.51 higher risk of developing MAEs compared to those who always double-check anesthetic drugs before administration (AOR = 3.51; 95% CI: 1.34, 9.19). Additionally, participants who administer medications that have been prepared by someone else are about five times more likely to experience MAEs than participants who prepare their own anesthetic medications prior to administration (AOR = 4.95; 95% CI: 1.54, 15.95) (Table 4).

**Table 4:** Underlying root causes of anesthetics drug administration error (n = 104)

Variables	Error		Odds ratio (95% CI)		Multi variate P value
	yes (n = 67)	no (n = 37)	Crude	Adjusted	
**Doubt**					
**yes**	18	19	1	1	
**no**	49	18	2.87(1.24, 6.66)*	2.45 (0.95, 6.33)	0.06
Double check drugs					
Yes	18	20	1	1	
No	49	17	3.20(1.38,7.44)*	3.51(1.34,9.19)	0.01
**Guidelines are accessible**					
**Yes**	18	15	1	1	
No	49	22	1.86(0.79, 4.34)*	1.41(0.51, 3.88)	0.50
**Who prepare anesthetics**					
**students under supervision**	32	11	1.01(0.35,2.91)*	0.97(0.30,3.13)	0.97
**another anesthetist**	23	8	4.36(1.6,11.88)*	4.95(1.54,15.95)	0.007
your own	12	18	1	1	
**how draw the drug into the srying**					
**withdraw the drug then label the srying**	37	14	2.03(0.89,4.60)	2.55(0.97, 6.72)	0.058
**label the syring first then withdraw the drug**	30	23	1	1	

## Discussion

The purpose of this study was to evaluate the frequency and underlying root causes of errors during medication administration among anaesthetists working in Amhara region, Ethiopia. The results of this study indicated that majority (64.4%) of anaesthetists had admitted MAEs. This result is comparable to the findings of a study from Saudi Arabia, which found that 69% of respondents had made at least one medication error while administering anaesthesia [15]. However, the study results from Santa Catarina, India and canada shows a higher rate of occurrence, where the authors reported that the prevalence of drug administration errors during anaesthesia practice among anesthesiologists is 91.8%, 75.6% and 85% respectively [12, 16, 17]. That might be because their study design was different from ours and they used direct observation to assess drug administration error in addition to a self-reported survey.

Even though Amponash et al. found that errors were more likely to occur during night shifts, our study found no correlation between shift of working time and MAEs. This could be as a result of that more than two thirds of participants said they worked an alternative shift with a similar caseload during both shifts. Additionally, this could be the result of the fact that fatigue brought on by a heavy workload is a significant contributing factor to MAEs regardless of the time of working shift [18]. Furthermore, this is supported by the result that shown medication errors are believed to occur at any time by the majority of respondents in a Saudi Arabian study [19].

Because of the multitasking nature of anaesthesia practise, it is possible for clinicians to misidentify a drug while attending to other tasks [20]. According to reports, the two most important preventive measures for MAEs are the use of color-coded syringes and double-checking the medication, which involves two people verifying the same information [7, 21]. However, nearly all participants in this study (97.11%) stated that their hospital does not have color-coded syringes. Also, 63.5% of anaesthetists don’t always double-check their medications before administration and almost half (49%) of the participants claimed they withdraw the medication before labelling the syringe. These factors could lead to incorrect syringe identification and, even worse, anaesthetists administering drugs incorrectly because they conflict with preventive standards for MAEs [11, 22]. This is supported by a related study from a hospital in China, where Zhang et al. reported that incorrectly labelled syringes are the root cause of syringe swapping, or the unintentional administration of the wrong medication. [23]. Additionally, inadequate syringe labelling was ranked by study participants as the third most important factor contributing to medication errors, behind a heavy workload and haste, according to the literature [20]. Instead, it is reported that two of the five strong evidence-based recommendations for minimising errors in the administration of intravenous anaesthetic drugs, labeling syringes always before administering drugs and double-checking labels with a second person using a formal organisation of drug drawers and workstations are the measures that lower MAEs [21].

Among participants who admitted MAEs, 51.92% did not report any drug administration related errors while they were at work. Despite the fact that this finding is alarming, it is consistent with earlier studies that have shown that most respondents who admitted to MAEs were unwilling to report even one drug error over the course of their careers [10, 14, 24]. Alternatively, it could be explained by scientific data showing that clinicians experience shame, guilt, or blame after disclosing their errors, which may discourage them from reporting errors [9–11].

When asked why they were reluctant to report their errors during medication administration process, 30.77% of respondents stated that they were concerned about the medicolegal repercussions of doing so. The final two reasons given by respondents as reasons why they were unable to disclose their errors were: not knowing to whom it was reported (24.04%) and fear of judgment by colleagues.

In response to a question, more than half of participants (53.8%) said that their hospital had no incident reporting system at all. Similarly, participants admitted they had no idea when or how their hospitals conducted the reported error audits. This finding might point to a lack of knowledge among clinicians regarding the hospital’s policies for reporting errors. It is consistent with earlier studies, where 23.9% of respondents had medication errors but only 6% were willing to admit to their errors [25].

In this study, participants who administer medications that have been prepared by someone else are about five times more likely to experience MAEs than participants who prepare their own anesthetic medications prior to administration. This may be the result of poor communication among anesthetists, as shown by the the study that only 35.57% of participants spoke to a colleague anesthetist when in doubt about drug administration process. This finding is in line with research by Leonard et al., which found that participants’ poor communication led to 48% of medication errors and 70% of medication error related adverse event [26].

The findings of this study showed that the presence of MAEs among the participants was significantly related to whether anaesthetists consistently double-check their anaesthetic drugs before administration. As a result, there was a 3.51 increase in the risk of MAEs among anaesthetists who did not always check their anaesthetic medications before administration. Among the potential safety advantages of double checking medications before administration is the reduction of endogenous (that originate from one person) and exogenous errors (that result from external factors, such as illegible text) [27]. our finding is supported by a randomized control trial from the United States, where multivariate regression revealed that double-checked administrations were significantly associated with a lower risk of any type of error [28].

## Conclusion

Our study found a considerable rate of errors in the administration of anaesthetic drugs. The failure to always double-check medications before administration and the use of drugs prepared by another anaesthetist were identified to be risk factors for drug administration errors. To ensure the safety of surgical patients, it is imperative to prioritise preventing these clinical errors. As a result, preparing anaesthetic medications on one’s own and double checking the drugs before administration will reduce the frequency of medication administration errors and enhance patient outcomes.

### Limitation

As a survey study, the study’s main limitation is that it relies on “self-report,“ which could have overstated or understated the frequency of drug admnistration errors. Despite the fact that our data show a significant incidence rate of anaesthetic medication errors, the sample size was too small to fully represent the entire anesthetists community. Therefore, for any relevant future investigations, we suggest undertaking a direct observational study with a larger sample size.

## List of Abbreviations

MAEs: Medication administration errors
